# General practice trainees’ telehealth use during the COVID-19 pandemic: a cross-sectional study

**DOI:** 10.1093/fampra/cmad022

**Published:** 2023-03-07

**Authors:** Katie Fisher, Amanda Tapley, Anna Ralston, Andrew Davey, Alison Fielding, Mieke van Driel, Elizabeth Holliday, Jean Ball, Jason Dizon, Neil Spike, Lisa Clarke, Parker Magin

**Affiliations:** University of Newcastle, School of Medicine and Public Health, University Drive, Callaghan, NSW, Australia; GP Synergy, Regional Training Organisation, NSW & ACT Research and Evaluation Unit, 20 McIntosh Drive, Mayfield West, NSW, Australia; University of Newcastle, School of Medicine and Public Health, University Drive, Callaghan, NSW, Australia; GP Synergy, Regional Training Organisation, NSW & ACT Research and Evaluation Unit, 20 McIntosh Drive, Mayfield West, NSW, Australia; University of Newcastle, School of Medicine and Public Health, University Drive, Callaghan, NSW, Australia; GP Synergy, Regional Training Organisation, NSW & ACT Research and Evaluation Unit, 20 McIntosh Drive, Mayfield West, NSW, Australia; University of Newcastle, School of Medicine and Public Health, University Drive, Callaghan, NSW, Australia; GP Synergy, Regional Training Organisation, NSW & ACT Research and Evaluation Unit, 20 McIntosh Drive, Mayfield West, NSW, Australia; University of Newcastle, School of Medicine and Public Health, University Drive, Callaghan, NSW, Australia; GP Synergy, Regional Training Organisation, NSW & ACT Research and Evaluation Unit, 20 McIntosh Drive, Mayfield West, NSW, Australia; University of Queensland, Faculty of Medicine, General Practice Clinical Unit, Level 8 Health Sciences Building, Royal Brisbane & Women’s Hospital, Brisbane, QLD, Australia; University of Newcastle, School of Medicine and Public Health, University Drive, Callaghan, NSW, Australia; Hunter Medical Research Institute, Clinical Research Design, IT and Statistical Support Unit (CReDITSS), New Lambton Heights, NSW, Australia; Hunter Medical Research Institute, Clinical Research Design, IT and Statistical Support Unit (CReDITSS), New Lambton Heights, NSW, Australia; Eastern Victoria General Practice Training, Regional Training Organisation, Hawthorn, VIC, Australia; University of Melbourne, Department of General Practice and Primary Health Care, Berkeley Street, Carlton, VIC, Australia; Monash University, School of Rural Health, Faculty of Medicine, Nursing and Health Sciences, Clayton, VIC, Australia; General Practice Training Tasmania, Regional Training Organisation, Hobart, TAS, Australia; University of Newcastle, School of Medicine and Public Health, University Drive, Callaghan, NSW, Australia; GP Synergy, Regional Training Organisation, NSW & ACT Research and Evaluation Unit, 20 McIntosh Drive, Mayfield West, NSW, Australia

**Keywords:** education, medical, graduate, family practice, general practice, telemedicine

## Abstract

**Background:**

Prompted by the COVID-19 pandemic, remuneration was introduced for Australian general practice telehealth consultations. General practitioner (GP) trainees’ telehealth use is of clinical, educational, and policy importance. The aim of this study was to assess the prevalence and associations of telehealth versus face-to-face consultations amongst Australian GP registrars (vocational GP trainees).

**Methods:**

Cross-sectional analysis of data from the Registrar Clinical Encounters in Training (ReCEnT) study, from 2020 to 2021 (three 6-month terms), including registrars in 3 of Australia’s 9 Regional Training Organisations. In ReCEnT, GP registrars record details of 60 consecutive consultations, 6 monthly. The primary analysis used univariate and multivariable logistic regression, with outcome of whether the consultation was conducted via telehealth (phone and videoconference) or face-to-face.

**Results:**

1,168 registrars recorded details of 102,286 consultations, of which 21.4% (95% confidence interval [CI]: 21.1%–21.6%) were conducted via telehealth. Statistically significant associations of a telehealth consultation included shorter consultation duration (odds ratio [OR] 0.93, 95% CI: 0.93–0.94; and mean 12.9 versus 18.7 min); fewer problems addressed per consultation (OR 0.92, 95% CI: 0.87–0.97); being less likely to seek assistance from a supervisor (OR 0.86, 95% CI: 0.76–0.96) while being more likely to generate learning goals (OR 1.18, 95% CI: 1.02–1.37); and being more likely to arrange a follow-up consultation (OR 1.18, 95% CI: 1.02–1.35).

**Conclusions:**

That telehealth consultations were shorter, with higher rates of follow-up, has GP workforce/workload implications. That telehealth consultations were less likely to involve in-consultation supervisor support, but more likely to generate learning goals, has educational implications.

**Key messages**

- We conducted an observational study of GP registrars’ telehealth use.
- There was low uptake of videoconferencing (3.4% of telehealth consultations).
- Telehealth consultations tend to be shorter with higher rates of follow-up.
- Trainees were less likely to seek supervisor support during telehealth consults.

## Introduction

Telehealth is defined as the delivery of health services over a distance via the use of telecommunication technologies. While telehealth exists in a variety of modalities, for the purpose of this paper we have focussed on videoconferencing and telephone consultations between doctors and patients.

In Australia, general practice is funded by a mixed model. This primarily involves Australia’s public insurance fund, the Medicare Benefits Schedule (MBS). In March 2020, in response to COVID-19, MBS remuneration was introduced for telehealth consultations.^1^ An uptake of general practitioner (GP) telehealth consultations, to 36% of remunerated consultations, followed in April 2020.^2^ Telehealth’s main pandemic-related benefit was decreased COVID-19 transmission risk, however, ease of access (with decreased travel expenses and employment interruptions) was a by-product, particularly for those in rural and remote regions.^3^

An Australian study published early in the pandemic found that telehealth was associated with GP, practice, and patient characteristics. Scott et al. found a strong positive association between larger practice size and telehealth use, a lower proportion of videoconferencing amongst older GPs (>55 years old), and a greater proportion of videoconferencing amongst GPs who saw more complex patients.^4^ Scott et al. also commented on the need for infrastructure and training support to enable greater facilitation of videoconferencing in general practice.^4^ Further, Javanparast et al. explored the experiences of Australian GP patients at high risk of poor health outcomes during the pandemic and found that participants were generally satisfied with telehealth.^5^ However, participants described challenges, including communication barriers and difficulty in accessing physical examination.^5^

At the time of writing, there is limited literature regarding vocational general practice trainees’ use of telehealth. There are various terms internationally for these vocational trainees, for example, often referred to as “trainees” in countries such as the United Kingdom, or “residents” in North America. In Australia, they are referred to as “registrars.” We will use the term “registrars” in this report.

Chaudhry et al. have reported on the experiences of GP registrars undertaking telephone consultations in the UK prepandemic,^6^ and Coenen et al. have reported on the impacts of COVID-19, including telehealth, on GP training in Belgium.^7^ These studies indicated that GP registrars find telephone consulting challenging, particularly related to limited physical examination and increased diagnostic uncertainty.^6,7^

Early-career GPs, including GP registrars are an important clinician group in considerations of telehealth uptake.^8^ GP registrars make up 11% of all Australian GPs by headcount,^9^ and will be taking telehealth technological changes into future practice. This study aims to address an evidence gap by establishing the prevalence and associations of Australian GP registrars’ telehealth use.

## Methods

### Study design

This exploratory study was a cross-sectional analysis of data from the Registrar Clinical Encounters in Training (ReCEnT) project.

### ReCEnT

ReCEnT is an ongoing (from 2010) multisite cohort study of the educational and clinical content of general practice registrars’ in-practice consultations.^10,11^

In Australia, GP registrars undergo at least three 6-month (full-time equivalent) terms in community-based general practice settings. Registrars train in an apprenticeship-like model wherein they have recourse to assistance and teaching from an experienced GP supervisor.^12,13^ However, they practice with considerable autonomy—conducting comprehensive general practice, with prescribing, test-ordering, and billing privileges equivalent to established GPs.

In ReCEnT, registrars record (via an online portal) details of 60 consecutive consultations each term as a routine component of their educational programme. Only in-practice consultations are included, with home visits and residential aged care facility visits excluded. Also excluded are in-practice consultations as part of single purpose “clinics,” for example immunization clinics.^11^ Registrars may also provide written consent to their data being used for research purposes.

During 2020 and 2021, ReCEnT was conducted in 3 GP vocational training Regional Training Organisations (RTOs) across 3 states (New South Wales, Victoria, and Tasmania) and the Australian Capital Territory. The 3 RTOs train 43% of Australian GP registrars.^14^

### Outcome factor

The outcome factor was telehealth versus face-to-face consultations. MBS item numbers were used to define telehealth (including both videoconference and telephone) consultations.^1^

### Independent variables

Independent variables included in analyses are presented in Table 1.

**Table 1.** Independent variables included in analyses.

Registrar variables	• Age • Gender • Training term • Place of medical qualification (Australia/international) • Full-time/part-time status • Aboriginal and/or Torres Strait Islander status • Whether the registrar had previously worked at the practice • Whether the registrar had prior health qualifications or post-graduate qualifications • Whether the registrar does work outside of GP training
Patient variables	• Age • Gender • Aboriginal and/or Torres Strait Islander status • Non-English-speaking background • Patient status: new to the practice (and thereby new to the registrar), new to the registrar (but existing patient of the practice), or existing patient of both the practice and registrar • Problem status of consultation (only old problems, only new problems, or both old and new problems)
Practice variables	• Practice region • Rurality/urbanicity, as determined by the Australian Statistical Geography Standard Remoteness Area (ASGS-RA)^15^ • Practice size (full-time equivalent GPs) • If the practice routinely bulk bills • Practice location’s Socio-Economic Indexes for Areas—Index of Relative Socioeconomic Disadvantage (SEIFA-IRSD)^16^
Consultation variables	• Number of problems addressed in the consultation • Consultation duration • Whether the consultation was performed in a language other than English • Whether assistance or advice was sought • Whether the consultation included a chronic disease management plan
Consultation action variables	• Medications prescribed • Imaging ordered • Pathology ordered • Follow-up organized • Referrals made • Learning goals generated

### Statistical analysis

We conducted a cross-sectional analysis of three 6-monthly rounds of data from 2020 to 2021. Proportions of consultations billed as telehealth were calculated with 95% confidence intervals (CIs). Descriptive statistics included frequencies for categorical variables and mean with standard deviation for continuous variables.

Associations of a consultation being billed as telehealth were analysed using univariate and multivariable logistic regression. Regressions were conducted within the Generalized Estimating Equations framework to adjust for clustering of consultations within registrars. An exchangeable working correlation structure was assumed.

Univariate analyses were conducted on each covariate, with the outcome. Multivariable analysis was conducted including all covariates of interest. Covariates with *P* values <0.20 in either univariate or multivariable analyses were considered for inclusion in the multiple regression model.

Once the model with all significant covariates was fitted, model reduction was assessed. Covariates which were no longer significant (at *P* < 0.2) in the multivariable model were tested for removal from the model. If the covariate’s removal did not substantively change the resulting model (any covariate in the model having a change in the effect size [odds ratio] of greater than 10%), the covariate was removed from the final model.

Effects were expressed as ORs with 95% CI. Statistical significance was declared at the conventional 0.05 level, with the magnitude and precision of effect estimates also used to interpret results.

To examine different facets of our study aims, 3 models were built, each with “consultation being via telehealth” as the dependent variable. To examine the associations of a consultation being conducted by telehealth rather than being face-to-face, patient, practice, and registrar independent variables were included in an initial multivariable regression model. To examine how consultations conducted via telehealth differ from face-to-face consultations, the above variables were included in a second multivariable model along with the 5 “consultation variables” listed in Table 1. To examine how outcomes of consultations conducted via telehealth compared with those of face-to-face consultations, all variables from the previous models were included in a final multivariable model along with the 6 “consultation action variables” listed in Table 1.

The rationale for building 3 models was that associations of a registrar’s consultation being conducted via telehealth will include patient, registrar, and practice factors, but evaluation of these associations may be compromised by inclusion in the multivariable model of factors operating once the consultation is progressing. Similarly, evaluation of the content of the consultation may be compromised by the inclusion in this model of outcomes arising from the consultation.

Statistical analyses were programmed using STATA 16.0 (StataCorp, College Station, TX) and SAS V9.4 (SAS Institute Inc., Cary, NC).

### Ethics approval

This study has ethics approval from the University of Newcastle Human Research Ethics Committee Reference H-2009-0323.

## Results

A total of 1,168 GP registrars (response rate 70.1%) recorded 102,286 encounters across three 6-month terms from 2020 to 2021. Of these encounters, 21,879 (21.4%, 95% CI: 21.1%–21.6%) were telehealth consultations (96.6% telephone [95% CI: 96.3%–96.8%] versus 3.4% videoconference [95% CI: 3.2%–3.7%]).

**Fig. 1.**
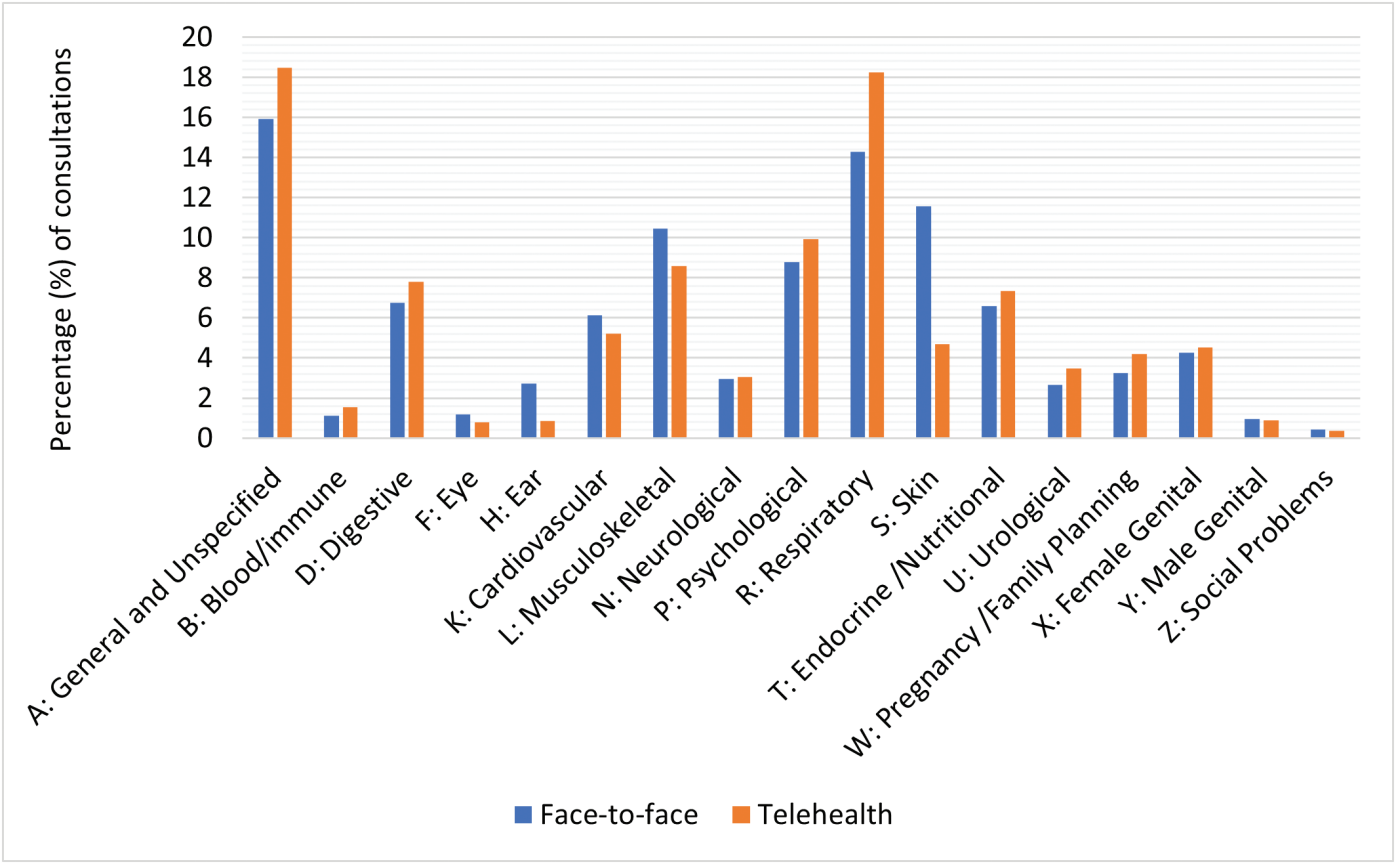
Percentage of consultations by International Classification of Primary Care-2 Disease Chapters for face-to-face versus telehealth (phone and video) (*n* = 102,286 consultations, 2020–2021).

Figure 1 demonstrates the percentage of consultations by International Classification of Primary Care 2nd edition (ICPC-2) Disease Chapters^17^ for telehealth versus face-to-face. Figure 1 shows that disease chapters such as respiratory and psychological made up a larger percentage of telehealth consultations, whereas chapters such as eye, ear, skin, and musculoskeletal made up a larger percentage of face-to-face consultations.

Registrar and practice demographics are shown in Table 2. Characteristics associated with telehealth versus face-to-face consultations are shown in Table 3, and the univariate and multivariable regression models are shown in Table 4.

**Table 2.** Demographics of participating GP registrars and their practices (*n* = 1,168 registrars, 2020–2021).

	Variables	Class	Total *n* (%)
Registrar characteristics (*n* = 1,168)	Registrar gender	Female	680 (58.3)
	Qualified as doctor in Australia	Yes	914 (80.2)
Registrar-round characteristics (*n* = 1,721)	Registrar age (years)	Mean (SD^a^)	32.9 (6.0)
	Registrar works full-time	Yes	1,174 (75.8)
	Registrar training term	Term 1	702 (40.8)
		Term 2	415 (24.1)
		Term 3	604 (35.1)
	Size of practice	Small (1–5 GPs)	604 (39.0)
		Large (6+ GPs)	946 (61.0)
	Practice routinely bulk bills	Yes	574 (36.9)
	Registrar worked at practice previously	Yes	378 (24.3)
	Rurality of practice	Major city	980 (57.0)
		Inner regional	584 (34.0)
		Outer regional/ remote/very remote	156 (9.1)
	SEIFA-IRSD^b^ decile of practice	Mean (SD^a^)	5.4 (2.8)

^a^Standard deviation.

^b^Socio-Economic Indexes for Areas—Index of Relative Socioeconomic Disadvantage.

**Table 3.** Descriptive characteristics of telehealth versus face-to-face consultations (*n* = 1,168 registrars, *n* = 102,286 consultations, 2020–2021).

Factor group	Variable	Class	In person	Telehealth	*P*
Patient factors	Patient age group	0–14	12,143 (15%)	2,383 (11%)	<0.001
		15–34	21,703 (27%)	6,299 (29%)	
		35–64	29,452 (37%)	8,959 (41%)	
		65–74	9,039 (11%)	2,194 (10%)	
		75+	7,970 (10%)	2,016 (9%)	
	Patient gender	Male	33,600 (42%)	7,825 (36%)	<0.001
		Female	46,714 (58%)	14,031 (64%)	
	Aboriginal and/or Torres Strait Islander status	No	67,520 (97%)	18,695 (98%)	<0.001
		Yes	1,776 (3%)	327 (2%)	
	NESB^a^	No	56,629 (90%)	16,019 (93%)	<0.001
		Yes	6,393 (10%)	1,271 (7%)	
	Patient/practice status	Existing patient	34,572 (43%)	11,390 (52%)	<0.001
		New to registrar	39,241 (49%)	10,133 (46%)	
		New to practice	6,568 (8%)	350 (2%)	
	Problem status	Only old problems	26,493 (33%)	11,191 (51%)	<0.001
		Only new problems	41,339 (51%)	8,629 (39%)	
		Both old and new problems	12,567 (16%)	2,056 (9%)	
Registrar factors	Registrar gender	Male	34,586 (43%)	8,607 (39%)	0.75
		Female	45,410 (57%)	13,204 (61%)	
	Registrar full-time or part-time	Part-time	17,474 (24%)	4,690 (24%)	0.013
		Full-time	54,833 (76%)	14,958 (76%)	
	Term	Term 1	32,730 (41%)	8,944 (41%)	<0.001
		Term 2	18,932 (24%)	5,777 (26%)	
		Term 3	28,745 (36%)	7,158 (33%)	
	Worked at practice previously	No	54,665 (75%)	15,290 (77%)	<0.001
		Yes	17,931 (25%)	4,550 (23%)	
	Qualified as doctor in Australia	No	16,392 (21%)	3,543 (17%)	0.036
		Yes	61,469 (79%)	17,797 (83%)	
	Has previous qualification	No previous qualification	41,936 (54%)	11,106 (52%)	0.50
		Previous qualification, health	11,483 (15%)	3,157 (15%)	
		Previous qualification, non-health	24,442 (31%)	7,077 (33%)	
	Has post-graduate qualification	No	55,893 (72%)	14,758 (69%)	0.13
		Yes	21,968 (28%)	6,582 (31%)	
	Registrar Aboriginal and/or Torres Strait Islander status	No	76,958 (99%)	21,175 (99.2%)	0.42
		Yes	903 (1%)	165 (0.8%)	
	Has other regular medical work	No	60,546 (83%)	17,059 (86%)	0.72
		Yes	12,133 (17%)	2,817 (14%)	
	Registrar age	Mean (SD^b^)	33.11 (6.25)	32.17 (5.33)	<0.001
Practice factors	Practice region	1	9,276 (12%)	2,503 (11%)	<0.001
		2	4,865 (6%)	1,412 (6%)	
		3	12,625 (16%)	5,898 (27%)	
		4	25,367 (32%)	5,997 (27%)	
		5	28,274 (35%)	6,069 (28%)	
	Practice size	Small	29,329 (41%)	6,420 (33%)	0.94
		Large	43,033 (59%)	13,293 (67%)	
	Practice routinely bulk bills	No	45,244 (62%)	13,128 (66%)	0.59
		Yes	27,317 (38%)	6,746 (34%)	
	Rurality	Major city	44,931 (56%)	13,285 (61%)	0.79
		Inner regional	27,856 (35%)	6,862 (31%)	
		Outer regional and remote	7,560 (9%)	1,732 (8%)	
	SEIFA-IRSD^c^ decile of practice	Mean (SD^b^)	5.31 (2.78)	5.65 (2.78)	0.74
Consultation factors	Consult was in other language	No	79,741 (99.3%)	21,751 (99.7%)	0.012
		Yes	523 (0.7%)	63 (0.3%)	
	Chronic management plan	No	79,378 (99%)	21,717 (99.3%)	<0.001
		Yes	1,029 (1%)	162 (0.7%)	
	Sought help any source	None	63,148 (79%)	18,213 (83%)	<0.001
		Supervisor	6,524 (8%)	1,105 (5%)	
		Other sources	10,735 (13%)	2,561 (12%)	
	Medication prescribed	No	38,228 (48%)	12,326 (56%)	<0.001
		Yes	42,179 (52%)	9,553 (44%)	
	Pathology ordered	No	62,662 (78%)	18,375 (84%)	<0.001
		Yes	17,745 (22%)	3,504 (16%)	
	Imaging ordered	No	70,601 (88%)	20,551 (94%)	<0.001
		Yes	9,806 (12%)	1,328 (6%)	
	Follow-up ordered	None	43,506 (54%)	12,987 (59%)	<0.001
		With themselves	33,403 (42%)	7,568 (35%)	
		Other GP in the practice	3,498 (4%)	1,324 (6%)	
	Referral ordered	No	65,982 (82%)	18,484 (84%)	<0.001
		Yes	14,425 (18%)	3,395 (16%)	
	Learning goals generated	No	34,276 (76%)	6,840 (74%)	0.42
		Yes	11,122 (24%)	2,351 (26%)	
	Consultation duration	Mean (SD^b^)	18.70 (10.26)	12.91 (7.42)	<0.001
	Number of problems	Mean (SD^b^)	1.45 (0.75)	1.28 (0.59)	<0.001

^a^Non-English-speaking background.

^b^Standard deviation.

^c^Socio-Economic Indexes for Areas—Index of Relative Socioeconomic Disadvantage.

**Table 4.** Associations of telehealth versus face-to-face consultations (*n* = 1,168 registrars, *n* = 102,286 consultations, 2020–2021).

Factor group	Variable	Class	Univariate		Adjusted	
			OR^a^ (95% CI)	*P*	OR^a^ (95% CI)	*P*
Patient, registrar, and practice factors						
Patient factors	Patient age group. Referent: 15–34 years	0–14 years	0.68 (0.65–0.73)	<0.001	0.70 (0.63–0.77)	<0.001
		35–64 years	1.05 (1.01–1.09)	0.019	1.03 (0.97–1.10)	0.32
		65–74 years	0.84 (0.79–0.90)	<0.001	0.83 (0.76–0.91)	<0.001
		75+ years	0.87 (0.81–0.93)	<0.001	0.87 (0.79–0.96)	0.005
	Patient gender. Referent: male	Female	1.22 (1.18–1.26)	<0.001	1.15 (1.09–1.21)	<0.001
	Aboriginal and/or Torres Strait Islander status	Yes	0.79 (0.71–0.89)	<0.001	0.78 (0.66–0.92)	0.003
	NESB^b^	Yes	0.78 (0.71–0.85)	<0.001	0.81 (0.73–0.90)	<0.001
	Patient/practice status. Referent: existing patient	New to registrar	0.78 (0.74–0.82)	<0.001	0.81 (0.76–0.87)	<0.001
		New to practice	0.20 (0.18–0.23)	<0.001	0.26 (0.22–0.31)	<0.001
	Problem status. Referent: only old problems	Both old and new problems	0.39 (0.36–0.41)	<0.001	0.41 (0.38–0.45)	<0.001
		Only new problems	0.55 (0.52–0.57)	<0.001	0.59 (0.55–0.63)	<0.001
Registrar factors	Registrar full-time or part-time. Referent: full-time	Part-time	0.73 (0.57–0.94)	0.013	0.95 (0.70–1.28)	0.72
	Worked at practice previously	Yes	0.67 (0.58–0.77)	<0.001	0.51 (0.42–0.60)	<0.001
	Registrar age		0.97 (0.95–0.98)	<0.001	0.98 (0.97–1.00)	0.058
Practice factors	Practice region. Referent: 1	2	1.00 (0.65–1.53)	0.99	1.50 (0.95–2.38)	0.083
		3	1.56 (1.11–2.18)	0.010	1.23 (0.87–1.74)	0.24
		4	0.75 (0.50–1.12)	0.16	0.59 (0.40–0.88)	0.010
		5	0.74 (0.52–1.07)	0.11	0.71 (0.50–1.00)	0.051
	Practice routinely bulk bills	Yes	1.07 (0.83–1.37)	0.59	1.35 (1.00–1.83)	0.053
Consultation factors (adjusted for patient, registrar, and practice factors)						
Consultation factors	Sought help from any source. Referent: none	Other sources	0.81 (0.76–0.87)	<0.001	1.06 (0.97–1.16)	0.22
		Supervisor	0.58 (0.53–0.64)	<0.001	0.86 (0.76–0.96)	0.011
	Consultation duration		0.90 (0.89–0.91)	<0.001	0.93 (0.93–0.94)	<0.001
	Number of problems		0.64 (0.62–0.66)	<0.001	0.92 (0.87–0.97)	0.002
Consultation action factors (adjusted for patient, registrar, practice, and consultation factors)						
	Medication prescribed	Yes	0.74 (0.70–0.77)	<0.001	0.66 (0.58–0.74)	<0.001
	Pathology ordered	Yes	0.69 (0.65–0.72)	<0.001	0.85 (0.74–0.98)	0.022
	Imaging ordered	Yes	0.50 (0.47–0.53)	<0.001	0.71 (0.60–0.83)	<0.001
	Follow-up ordered	Other GP in the practice	1.13 (1.05–1.23)	0.002	1.33 (1.05–1.68)	0.019
		With themselves	0.76 (0.73–0.79)	<0.001	1.18 (1.02–1.35)	0.025
	Referral ordered	Yes	0.86 (0.82–0.90)	<0.001	1.18 (1.03–1.36)	0.019
	Learning goals generated	Yes	0.96 (0.88–1.05)	0.42	1.18 (1.02–1.37)	0.029

^a^Odds ratio.

^b^Non-English-speaking background.

On multivariable analysis, there were several statistically significant variables associated with telehealth.

For patient factors, registrars were less likely to see via telehealth those aged 0–14 years (adjusted odds ratio [aOR] 0.70, 95% CI: 0.63–0.77), 65–74 years (aOR 0.83, 95% CI: 0.76–0.91), and 75+ years (aOR 0.87, 95% CI: 0.79–0.96), compared with patients aged 15–34 years. Using telehealth, registrars were significantly more likely to see female patients (aOR 1.15, 95% CI: 1.09–1.21) but were less likely to see culturally and linguistically diverse (CALD) patients (aOR 0.81, 95% CI: 0.73–0.90) and Aboriginal and/or Torres Strait Islander patients (aOR 0.78, 95% CI: 0.66–0.92). Telehealth consultations were less likely for patients new to the registrar and new to the practice (aOR 0.81, 95% CI: 0.76–0.87 and aOR 0.26, 95% CI: 0.22–0.31, respectively). Telehealth was more likely for addressing only old problems (aOR 0.59, 95% CI: 0.55–0.63 for consultations addressing only new problems, aOR 0.41, 95% CI: 0.38–0.45 for both old and new problems).

For consultation factors, telehealth consultations were significantly shorter in duration, averaging (unadjusted) 12.9 (SD 7.4) min versus 18.7 (SD 10.3) min for face-to-face (aOR 0.93, 95% CI: 0.93–0.94, for each additional minute), and addressed fewer problems per consultation, (unadjusted) 1.3 (SD 0.6) problems compared with 1.5 (SD 0.8) for face-to-face (aOR 0.92, 95% CI: 0.87–0.97). Registrars were also less likely to seek help from their supervisors during telehealth consultations (aOR 0.86, 95% CI: 0.76–0.96).

For consultation action variables, for telehealth consultations, medications were less likely to be prescribed (aOR 0.66, 95% CI: 0.58–0.74), pathology was less likely to be ordered (aOR 0.85, 95% CI: 0.74–0.98), and imaging was less likely to be ordered (aOR 0.71, 95% CI: 0.60–0.83). Registrars were more likely to generate learning goals during telehealth consultations (aOR 1.18, 95% CI: 1.02–1.37). Follow-up appointments were more likely to be arranged following telehealth consultations, either with another GP in the practice (aOR 1.33, 95% CI: 1.05–1.68) or with the registrar themselves (aOR 1.18, 95% CI: 1.02–1.35). Referrals were more likely to be arranged during telehealth consultations (aOR 1.18, 95% CI: 1.03–1.36).

## Conclusions

### Summary

During 2020–2021, telehealth accounted for 21.4% of consultations performed by GP registrars. Of telehealth consultations, 3.4% were via videoconference. Telehealth consultations were less likely for Aboriginal and/or Torres Strait Islander patients, patients aged 0–14 and ≥65 years, and CALD patients. Telehealth consultations were shorter, addressed fewer problems per encounter, and had higher rates of follow-up. In addition, registrars were more likely to create learning goals, though less likely to seek in-consultation assistance from their supervisors.

### Strengths and limitations

The strengths of this study include a large sample size and high response rate^18,19^ from training practices in metropolitan, regional, and remote communities. There is strong generalizability to GP registrars across Australia. The generalizability to other countries, however, is uncertain.

A limitation of the cross-sectional analysis is an inability to establish causality in these associations.

### Comparison with existing literature

Despite not being entirely comparable, GP registrars had a lower uptake of telehealth compared with nationwide Australian GPs over a similar period (37%).^20^ This may reflect lesser confidence in performing telephone consultations, which has been reported amongst UK GP registrars prepandemic.^6^ GP registrars, however, had higher videoconference uptake compared with nationwide data (2.4%).^20^ This may reflect the younger age of GP registrars (mean age 32.9 years in this analysis compared with GP Fellows, of whom two-thirds are aged ≥45 years old)^21^ and consequent greater facility with digital applications and videoconferencing technology. Video consultations have previously been found less likely to be used by GPs over 55 years.^4^ Our findings of a lack of association with telehealth use with registrar age, gender, hours worked, or overseas training are also consistent with findings in unselected GPs.^4^

Our findings of telehealth consultations being shorter, and addressing fewer problems per consultation are consistent with previous research.^22,23^ However, our analysis also found that GP registrars were more likely to schedule follow-up appointments for patients following telehealth consultations, which could increase GP workload. This has been commented on in other contexts, with increased presentations following the use of telephone triage^24^ and telephone consultations for same-day appointments.^23^ However, it is worth noting that many general practices in Australia, during the pandemic, were exclusively offering telehealth appointments before booking a face-to-face review to reduce COVID-19 transmission risk, which could explain these findings. In addition, it has been widely reported that chronic care and nonurgent medical issues were being seen less during the pandemic,^25–27^ which could have resulted in shorter appointments.

Female patients were more likely to use telehealth compared with males, as noted in other telehealth studies.^22,28^ Australian men are less likely to have a regular general practice,^29^ which may have been a barrier to telehealth given the eligibility requirements (having to be seen face-to-face at the practice, or with the medical practitioner, in the preceding 12 months).^1^

Patients aged 0–14 years, and those aged 65 years and older, were less likely to use telehealth compared with other age groups, which is comparable to other studies.^22,28^ Elderly patients may experience barriers to telehealth, particularly for videoconsultations, including lack of familiarity with technological applications and lack of access to digital devices.^30^ Hearing and visual impairments can also pose challenges to telehealth uptake in this cohort.^31^ Improving access for the elderly needs to be considered in future telehealth models, particularly as this cohort experience a higher burden of chronic disease^32^ and considerable barriers to accessing health services.^33^

Aboriginal and/or Torres Strait Islander and CALD patients were less likely to use telehealth compared with non-Indigenous patients and native English speakers, respectively. This is comparable to several American studies, which found that non-English-speaking and non-White patients were less likely to use videoconferencing.^34–36^ However, this is in contrast to a systematic review, prepandemic, which found that Aboriginal and/or Torres Strait Islanders reported positive interactions with telehealth and improved access to quality health services.^37^ This suggests that, whilst Aboriginal and/or Torres Strait Islanders and CALD patients may benefit from telehealth, there are potential barriers to its use, likely due to an amplification of existing language, communication, and cultural barriers. Another consideration could be socioeconomic factors in these vulnerable population groups, affecting internet and phone access to support telehealth consultations.^3^

GP registrars were less likely to seek help from their supervisors, or any other sources (electronic resources, books, etc.), during telehealth consultations. This may be, in part, due to the logistical difficulty of keeping a patient on the phone while waiting for a supervisor. It is possible that registrars sought advice from their supervisors at the end of their consulting days to mitigate this (noting that generation of learning goals for post-consultation review was more common during telehealth consultations). This has important implications for adequate supervision when registrars are performing telehealth consultations. It is particularly relevant as Chaudhry et al. found that GP registrars in the UK, prepandemic, felt least confident in independently undertaking complex aspects of telephone consulting.^6^ Negative experiences were reported with complex clinical scenarios (such as patients with multiple comorbidities), communication barriers, and inability to physically examine patients,^6^ which are all important components of registrar learning. Notably, there was a positive correlation between training received and registrar confidence,^6^ highlighting the value of providing telehealth-specific training to GP registrars.

Registrars were less likely to prescribe medications and order imaging during telehealth consultations. This has been reported by McKinstry et al. in their telehealth study,^23^ and Wabe et al. who found lower medication prescribing in telehealth consultations, compared with face-to-face, in Australian general practice.^38^ However, this result was somewhat unexpected, as we anticipated that telehealth would be commonly used for repeat prescriptions, which do not usually require an in-person visit. For imaging ordering, it is possible that registrars followed up patients face-to-face to physically examine them prior to arranging investigations, which would align with best practice.

### Implications for future research and practice

That telehealth consultations were shorter, with higher rates of follow-up, along with the observation that telehealth has increased patient access—there being less barriers to booking appointments for minor problems^39^—has GP workload/workforce implications. An Australian qualitative study found that medical practitioners felt efficiency could be improved by triaging patients to telehealth versus face-to-face, to reduce delays in patient care.^40^ Optimal practice systems for judicious triage may balance potentially competing factors of GP workload and service availability. Further research is needed to determine the most appropriate, and time-efficient, process for triaging to telehealth versus face-to-face.

Shorter consultations raise implications for registrar training, highlighting the different profile of clinical encounters that registrars are likely exposed to via telehealth (though noting a potential effect on consultation duration of chronic care and nonurgent medical issues being seen less during the pandemic^25–27^). Medical educators and teaching practices will need to consider the impacts of telehealth on the diversity of registrar encounters and ensure appropriate face-to-face caseloads are maintained during training. This is also important when considering physical examination skills, with less opportunity to practice these via telehealth. Further research is needed to address the impact of telehealth on GP training beyond the COVID-19 pandemic.

The low proportion of consultations performed via videoconferencing may represent a missed opportunity to perform (limited) physical examination and respond to patients’ nonverbal cues. GP registrars would benefit from telehealth training early in their first general practice term, with particular attention to performing a remote physical examination. This is a relatively new form of examination that is unlikely to have been taught prior to commencing GP training. Policymakers should consider supporting more accessible encrypted videoconferencing systems, and increasing remuneration for videoconsultations compared with phone, to target the low video uptake.

Lastly, our findings raised implications for possible barriers to telehealth experienced by vulnerable population groups, including the elderly, Aboriginal and/or Torres Strait Islander people, and CALD communities. Further research is needed to explore these barriers and identify potential solutions to increase telehealth access. This will be relevant for policymakers to consider when designing future telehealth models, with consideration of interpreter-integrated telehealth services, increased accessibility to encrypted videoconferencing platforms, and telehealth training for both GPs and patients.

## Supplementary Material

cmad022_suppl_Supplementary_Checklist

## Data Availability

The data that supports this study cannot be publicly shared due to ethical or privacy reasons.
